# An ex vivo human cartilage repair model to evaluate the potency of a cartilage cell transplant

**DOI:** 10.1186/s12967-016-1065-8

**Published:** 2016-11-15

**Authors:** Christoph Bartz, Miriam Meixner, Petra Giesemann, Giulietta Roël, Grit-Carsta Bulwin, Jeske J. Smink

**Affiliations:** co.don® AG, Biotechnology and Tissue Engineering, Teltow, Germany

**Keywords:** ATMP, Cartilage repair, Cell therapy, Autologous chondrocyte implantation, Potency, Regeneration, Cartilage condyle chip assay, Aggrecan

## Abstract

**Background:**

Cell-based therapies such as autologous chondrocyte implantation are promising therapeutic approaches to treat cartilage defects to prevent further cartilage degeneration. To assure consistent quality of cell-based therapeutics, it is important to be able to predict the biological activity of such products. This requires the development of a potency assay, which assesses a characteristic of the cell transplant before implantation that can predict its cartilage regeneration capacity after implantation. In this study, an ex vivo human cartilage repair model was developed as quality assessment tool for potency and applied to co.don’s chondrosphere product, a matrix-associated autologous chondrocyte implant (chondrocyte spheroids) that is in clinical use in Germany.

**Methods:**

Chondrocyte spheroids were generated from 14 donors, and implanted into a subchondral cartilage defect that was manually generated in human articular cartilage tissue. Implanted spheroids and cartilage tissue were co-cultured ex vivo for 12 weeks to allow regeneration processes to form new tissue within the cartilage defect. Before implantation, spheroid characteristics like glycosaminoglycan production and gene and protein expression of chondrogenic markers were assessed for each donor sample and compared to determine donor-dependent variation.

**Results:**

After the co-cultivation, histological analyses showed the formation of repair tissue within the cartilage defect, which varied in amount for the different donors. In the repair tissue, aggrecan protein was expressed and extra-cellular matrix cartilage fibers were present, both indicative for a cartilage hyaline-like character of the repair tissue. The amount of formed repair tissue was used as a read-out for regeneration capacity and was correlated with the spheroid characteristics determined before implantation. A positive correlation was found between high level of aggrecan protein expression in spheroids before implantation and a higher regeneration potential after implantation, reflected by more newly formed repair tissue.

**Conclusion:**

This demonstrated that aggrecan protein expression levels in spheroids before implantation can potentially be used as surrogate potency assay for the cartilage cell transplant to predict its regenerative capacity after implantation in human patients.

**Electronic supplementary material:**

The online version of this article (doi:10.1186/s12967-016-1065-8) contains supplementary material, which is available to authorized users.

## Background

Excessive activity or weight, traumatic accidents or congenital abnormalities can lead to damaged and injured articular cartilage [1]. However, articular hyaline cartilage has a low intrinsic repair capacity due to its lack of blood and lymphatic vessels as well its sparse cell population. Therefore, articular cartilage defects require early treatment before progression into osteoarthritis (OA) [2].

Currently, there are different treatment options for cartilage defects, depending on defect size and the state of the subchondral bone. Cartilage defects can be treated by bone marrow stimulating techniques, by osteochondral autograft transplantation or by implantation of cells and/or scaffolds [3]. Cell therapeutics to treat cartilage defects, include autologous chondrocyte implantation (ACI) products. ACI is a two-step surgical procedure using implantation of autologous cells to repair cartilage defects. In the first step, autologous chondrocytes are harvested from a biopsy of a non-weight bearing area of the cartilage of the patient. After cell expansion in vitro, the cells are re-implanted by different methods into the cartilage defect [4]. Cells can be injected as cell suspension or be matrix-associated. Matrix-associated ACI’s (M-ACI) can either consist of cells seeded onto artificial matrices derived from xenogenous biomaterials as collagen type I/III or they consist of autologous cells encapsulated in an endogenously produced extracellular matrix (ECM) forming spherical aggregates (chondrocyte spheroids) as in the product chondrosphere [3, 5]. These spherical aggregates develop cell–cell and cell–matrix contacts and thereby induce the synthesis of their own ECM [6, 7].

A major challenge of cell-based products is to fulfill critical quality parameters to ensure a consistent quality of the product and thereby a consistent clinical effect [8]. These critical quality attributes include: identity, purity, efficacy, safety and potency of the cell transplant, requiring the development of assays that can assess these attributes [9, 10]. Potency of cell transplants should be able to predict the biological activity of the product after implantation, based on the measurement of a specific characteristic of the transplant before implantation. Such assays must measure biological activities and functions of the product, including relevant indication-specific key mechanisms [8, 10]. Although in vivo animal models can be used to assess potency [11], animal models can be replaced by in vitro or ex vivo models [8, 11].

A potency assay for ACI products should include the assessment of a characteristic of the cartilage cell transplant before implantation that can predict their cartilage regeneration capacity after implantation. A set of several biological functional assays is suggested, including the quantitative analysis of the production of bioactive molecules, e.g. glycosaminoglycans (GAGs) or growth factors using different methods [9, 12, 13]. Developing and establishing potency assays of ACI products are still complicated and complex, due to unknown cellular processes (e.g. de- and redifferentiation) during the manufacturing process or after implantation [14].

co.don^®^ AG’s ACI product chondrosphere is a matrix-associated autologous chondrocyte implant that is in clinical use in Germany. Our strategy to establish a potency assay for chondrosphere is based on previous studies using cartilage chip assays to assess regeneration. First of all, an ex vivo cartilage repair model using bovine cartilage tissue demonstrated tissue regeneration capacity of implanted bovine chondrocytes ex vivo [15]. In addition, implanting human bone-marrow derived stem cells (hBMSC) in alginate using the same bovine osteochondral cartilage chips and subsequent subcutaneous implantation in nude mice demonstrated the formation of repair tissue within the defect after 4 weeks [16]. Secondly, in a previously performed preclinical in vivo experiment addressing the potency of chondrosphere, human cartilage condyle chips implanted with chondrosphere were subcutaneously implanted in nude mice to demonstrate the regeneration capacity of chondrosphere and the formation of repair tissue with a hyaline-like cartilage character could be demonstrated [7]. However, in this preclinical study the spheroids were not characterized before implantation and therefore no correlation could be assessed between spheroids characteristics before implantation and their regenerative capacity after implantation and potency of the implants could not be assessed.

The aim of the current study was to develop a human-based potency assay using an ex vivo human cartilage repair model as a quality assessment tool, in order to determine potential chondrocyte spheroid characteristics that correlate to the regeneration capacity of chondrosphere after implantation. Chondrocyte spheroids derived from different donors were implanted into subchondral cartilage defects set into human cartilage condyle chips, reflecting human cartilage defects as they are treated in the clinic with chondrosphere. After 12 weeks of co-cultivation (ex vivo), the amount of regenerated tissue in the cartilage defects was used as regeneration capacity read-out. Regeneration capacity of the spheroids was subsequently correlated with the spheroid characteristics that were analysed before implantation, as production of cartilage-specific ECM components and expression of chondrogenic marker genes.

## Methods

### Study design

**Fig. 1:**
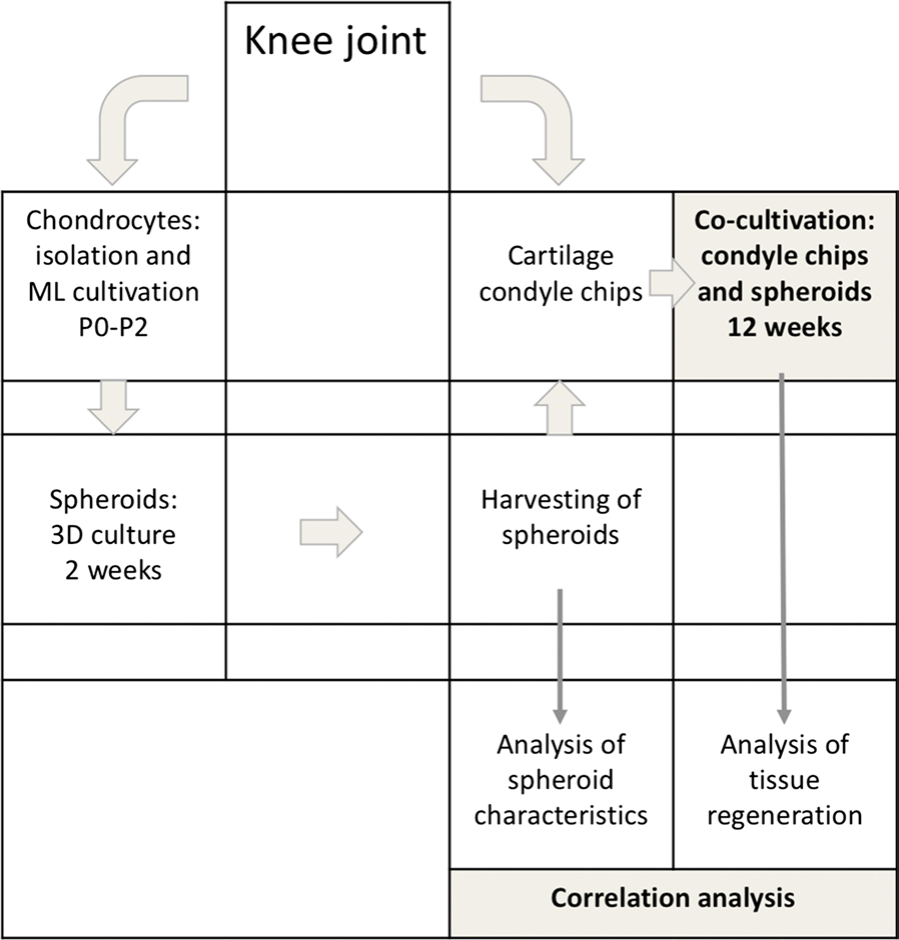
Flow chart of the ex vivo potency study. Schematic overview and work flow of the ex vivo experiments performed to establish a potency assay for the matrix-associated autologous chondrocyte implant chondrosphere. Human knee joints were used to generate both chondrocyte spheroids and the cartilage condyle chips used as cartilage defect model. Spheroids were analysed before implantation by means of histology, immunohistochemistry, GAG production and expression of chondrogenic marker genes. Spheroids derived from the same batch as characterised before implantation, were used to implant into subchondral cartilage defects set into the condyle cartilage chips, reflecting cartilage defects as treated in the clinic with chondrosphere. After co-cultivation of spheroids with the cartilage condyle chips, the level of tissue regeneration was quantified by histomorphometry. The regenerated tissue was furthermore analysed for hyaline cartilage specific protein expression. A correlation between the spheroid characteristics before implantation and the amount of regenerated tissue formed after 12 weeks of co-cultivation was statistically tested by calculating the Spearman’s correlation coefficient (r)

In order to develop a functional potency assay for chondrocyte spheroids, residing knee joint samples were processed for the generation of cartilage condyle chips as model system for cartilage defects and for isolation of chondrocytes to produce the chondrocyte spheroids for this study (Fig. 1). From every produced spheroid batch, a representative part of the spheroids was subject to analysis of specific proteins and/or gene expression before implantation, and a representative part of the same batch of spheroids were implanted in the condyle chips. After subsequent co-cultivation of the spheroids in the cartilage condyle chip for 12 weeks, regeneration of cartilage tissue was addressed using different methods. Assessment of correlation between regeneration potential and molecular characteristics of the spheroids before implantation will identify a possible potency marker that can be used to predict regeneration potential of the spheroids before implantation.

### Tissue material

Cartilage samples from 19 donors were obtained from biopsies of residing joint samples of donors who underwent surgery for knee endo-prosthesis. Samples were processed within 24 h after surgical removal to isolate the chondrocytes for spheroid production and/or generation of condyle chips (see description below). In addition, 4 spheroid samples were derived from the manufacturing department of codon® AG. To perform the chondrocyte and spheroid cultures, tissue of 14 different patients was used to generate spheroids and to generate the condyle chips, cartilage tissue of 16 different patients was used. All donor-derived material was used after written consent of the donor.

### Chondrocyte and spheroid culture

Healthy cartilage tissue was removed from the residing knee joint samples and minced. The chondrocytes were released from the cartilage tissue by enzymatic digestion using 0.25 U/ml collagenase at 37 °C overnight. Cells were cultured in cell culture medium supplemented with 10% human pool serum, no growth factors, antibiotics or other supplements were added. The human serum was derived from the manufacturing department and was tested on the presence of several viruses. The individual sera were tested for HIV, Hepatitis B and C as well as Syphilis. Serum of at least 15 patients was pooled. To produce spheroids, 2 × 10^5^ cells of third passage chondrocytes were seeded on coated non-adherent 96-well-plates [6]. The spheroids were cultured for 14 ± 1 days at 37 °C in a humidified atmosphere and 5% CO_2_. This also included spheroids from 4 donor samples originating from the manufacturing department of codon® AG.

After 14 ± 1 days of spheroid cultivation, each batch of a single donor was divided with one representative part of the spheroids being analysed for spheroid characteristics before implantation and the other representative part being implanted into the human artificial subchondral cartilage defects (see description below). This allowed for a direct comparison of spheroid characteristics before implantation and their potency to regenerate tissue after implantation.

### Preparation of cartilage condyle chips

The source for cartilage condyle chips was the same human knee joints as used to isolate chondrocytes for spheroid production. Condyle chips are a small solid piece of knee joint tissue consisting of cartilage and bone that were prepared to simulate cartilage defects as observed in the clinic, which can be treated with the cartilage cell transplant chondrosphere.

These so-called condyle-chips were cut in a defined size of at least 1 cm^2^ and subsequently stored in cell culture medium without additives at 4 °C (average storage time was 23 days) until 24 h before implantation. One day before spheroid implantation, subchondral cartilage defects (Ø 4 mm, until tidemark) were set manually in the condyle chips using a Ø 4 mm biopsy punch, thus standardized for every condyle chip. In the clinic, cartilage defects are also prepared and cleaned until the tidemark before implantation of the cartilage cell transplants. The prepared condyle chips were stored overnight at 37 °C and 5% CO_2_ covered with medium supplemented with 10% pooled human serum until implantation of the spheroids the next day.

### Co-cultures of implanted spheroids and cartilage condyle chips

The number of spheroids used for implantation in this study, was similar to the middle dosage of the matrix-associated autologous chondrocyte implant chondrosphere of 35 spheroids/cm^2^ in clinical practice (dose range of 10–70 spheroids/cm^2^). In the defect of Ø 4 mm, 5 spheroids were implanted. In condyle chips derived from 6 donors, a defect was set but no spheroids were implanted and these empty subchondral cartilage defects served as negative controls. Each co-culture of each individual donor was performed in duplicate. The term ‘co-culture’ was used in this study to indicate the interplay between the implanted spheroids and the cartilage tissue.

Condyle chips were transferred into 6-well plates and the required numbers of spheroids were implanted onto the dry defect ground. Spheroids were left to adhere at the base of the defect by incubation for 5–10 min at 37 °C and 5% CO_2_. Subsequently, the co-cultures were gently covered with cell culture medium supplemented with 10% pooled human serum and cultured at 37 °C and 5% CO_2_ for 12 weeks. Medium was exchanged twice a week for 75%. A total of 14 co-cultures were performed.

### GAG analysis

The amount of both released and bound sulfated glycosaminoglycans (GAG) in the cartilage cell transplants of individual spheroids were measured as described previously [17, 18]. The GAG produced and released into medium during the last 4 days of cultivation (from last medium exchange until implantation) were determined.

To detect GAG released into the medium and bound within the respective spheroid, the GAG assay according to Hoemann was performed [17]. To measure GAGs released into the medium, 30 µl medium of each sample was dispensed into a single well of a 96-well plate, and 60 µl of DMMB (1,9 Dimethyl Methylene Blue) was added to each well and incubated for 10 min at RT. The media samples were measured in triplicates. To quantify the GAG content in the spheroids, they were first digested with 100 µl papain-digestion buffer (35–45 U/spheroid, Sigma-Aldrich, 10 mM cysteine, 100 mM Na_3_PO_4_ buffer, 10 mM Na_2_EDTA) at 60 °C for 4 h with agitation (700 rpm). After digestion, the lysates were centrifuged for 5 min at 8000*g* at RT and 100 µl PBE with EDTA was added to the samples. 30 µl of the lysates of each sample was dispensed into a single well of a 96-well plate, and 40 µl of DMMB added to each well and incubated for 10 min at RT. All samples were measured in quadruplicates.

The amount of GAGs was quantified by measuring the ratios of absorbance at 525 and 595 nm using a plate reader (SpectraMax M2 microplate reader, Molecular Device). Shark chondroitin-6-sulfate (Sigma-Aldrich) was used to generate standard curves.

The ratio of bound/released GAG per spheroid was calculated by dividing the amount of GAGs bound within a respective spheroid and the amount of GAGs secreted by the same spheroid. A ratio of >1 indicated more GAGs bound into the spheroid than released into the medium by the respective sample.

### RNA isolation and qPCR

Total RNA of spheroids was isolated after 14 ± 1 days of spheroid cultivation (in two cases, #14 and #18, after 24 days) using the RNeasy Plus Mini Kit (Qiagen) according to the manufacturer’s instructions. cDNA was synthesized using the Transcriptor First Strand cDNA Synthesis Kit (Roche, Germany) according to the instructions of the manufacturer using 40–200 ng of RNA in the reverse transcription reaction. The gene expression analyses of the chondrogenic marker genes *CTRAC1* (cartilage acidic protein 1), *S100B* and *ACAN* (aggrecan) was performed by qPCR using the primer-UPL probe system of Roche (http://www.roche-applied-science.com/) and conducted on the LightCycler® 480 (Roche). Expression of *GAPDH* was used to normalize individual mRNA expression levels. The data are expressed as relative RNA expression levels and calculated using the comparative CT method (Roche). Sequences of primer pairs used can be obtained upon request.

### Histological and immunohistochemical analysis

For histological and immunohistochemical analysis, spheroids were fixed 1 h in buffered 4% formaldehyde at 4 °C and embedded in paraffin according to standard procedures. The condyle chips were fixed overnight in buffered 4% formaldehyde at 4 °C and subsequently decalcified for 3–5 weeks in 0.45 M phosphate-buffered EDTA, with the decalcification solution being refreshed two times per week. After decalcification, the defects were separated in two equal halves and embedded in paraffin according to standard procedures. Sections of 4 µm were cut and stained with Haematoxylin and Eosin (HE) for general morphological analysis, with Alcian blue for the detection of GAGs or to perform immunohistochemical stainings to detect the protein expression of specific cartilage markers.

Immunohistochemical stainings were performed as followed. Endogenous biotin binding sites were blocked with a biotin-blocking system (Dako) and additional non-specific binding was blocked with donkey serum (1:20 in 0.5% BSA/0.02% Tween/50 mM Tris pH 7.5). Antigen retrievals were performed by microwave treatment in 10 mM citrate buffer pH 6.0 for collagen type I (COL I) and aggrecan (ACAN) or for collagen type II (COL II) by treatment with hyaluronidase (15 min, 2.0 mg/L, pH 5.5) and pronase (30 min, 1.4 mg/L, pH 7.4). Collagen type I protein was detected using mouse anti-collagen type I (AM31694PU-N; Acris) in a dilution of 1/100, collagen type II protein was detected using mouse anti-collagen type II (63171) (MP Biomedicals) in a dilution of 1/1000 and aggrecan protein was detected using mouse anti-aggrecan (SM1353P; Acris) in a dilution of 1/200. The primary antibody was incubated at 4 °C overnight in a humidified chamber and as a negative control, an isotope control antibody in the same dilution as the primary antibody was used (mouse IgG, Sigma-Aldrich). For detection, the avidin–biotin-complex method followed by the Dako®Substrate chromogen system was used. Sections were counterstained with haematoxylin. Pictures were taken using an Axioskop 2 Plus microscope with an AxioCam MRc5 camera and Zen 2012 SP2 software (Zeiss).

### Histomorphometric analysis of formed repair tissue and correlation to spheroid characteristics

The extent of newly formed tissue in the human subchondral cartilage defects was assessed as area of repair tissue (RT) present within the defects after 12 weeks of co-cultivation. Histomorphometrical analyses on histological sections with interactive measurements using the software Zen 2012 SP2 from Zeiss enabled quantification of the newly formed tissue. These measurements excluded the areas occupied by the implanted spheroids and thus only reflected the new tissue formed. Additionally, the total defect area was measured to calculate the percentage area of newly formed tissue that filled the total defect area for each donor sample. Histomorphometrical analyses were performed in duplicate for each condyle chip. The newly formed tissue as percentage of total defect area was used as read-out parameter for the potency of the implanted spheroids.

Before implantation, the individual characteristics of the spheroids were analysed and scored. After scoring, the results of all donor samples were used to form a ranking from high to low levels of the specific parameter. Spheroid characteristics that were analysed included GAG production (both bound in the spheroid as well as released into the cell culture medium as well as the GAG ratio) and expression of chondrogenic markers at both the mRNA level as well as the protein level. The ranking of the individual spheroid characteristics of the individual donors was compared to the percentage newly formed tissue of the same donor.

### Statistical analysis

The obtained data are expressed as mean ± standard deviation. Spearman’s correlation coefficient was calculated as non-parametric measure for statistical dependence between two variables (spheroid characteristic and tissue regeneration). A *p* value of less than 0.05 was considered statistically significant. Statistical analyses and graphical design were performed using GraphPad Prism v6.0. Sample size was calculated to detect a difference (>1, 5× increase of regenerated tissue) between the negative control (empty condyle chip) and a treated sample (condyle chip with spheroids) with a power of 95% (β = 0.05), and α = 0.05 (http://www.clincalc.com/Stats/SampleSize.aspx).

## Results

### Tissue regeneration in a human cartilage repair model after implantation of spheroids

**Fig. 2:**
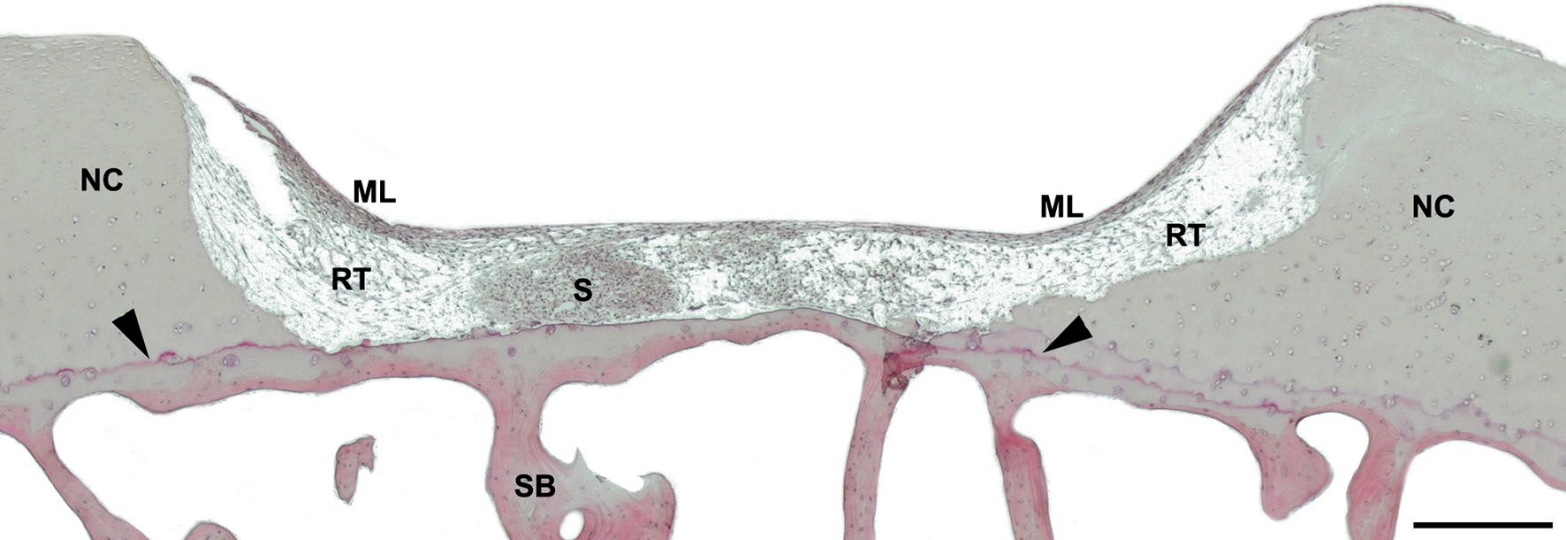
Formation of repair tissue after 12 weeks of co-cultivation. Histological appearance of repair tissue formed after 12 weeks of co-cultivation with implanted spheroids of a co-culture of patient #8. HE stained cross section of a standardized subchondral cartilage defect until tidemark (*arrowhead*) showing the presence of a disintegrated implanted spheroid (S). Newly formed regenerative tissue (RT) covered by a newly formed multilayer of cells (ML). The formed repair tissue is well integrated in the neighbouring native cartilage (NC). *Scale bar* 500 µm. *ML* multilayer, *NC* native cartilage, *S* spheroid, *SB* subchondral bone, *RT* regenerative tissue

Spheroids were implanted into human subchondral cartilage defects in cartilage condyle chips ex vivo, reflecting cartilage defects as treated in the clinic with a dosage 35 spheroids/cm^2^ of the matrix-associated autologous chondrocyte implant chondrosphere. After 12 weeks of co-cultivation of the spheroids and the cartilage condyle chips, the spheroids being present at the bottom of the defect showed nearly the same morphology (e.g. round–oval shape) as at the time of implantation in most of the donor samples. However, in two cases (donor #7, #12) spheroids were flattened at the defect ground (data not shown), and in two samples (donor #8, #9), the edges of spheroids were disintegrated in different stages and single cells were detached from the spheroid (Fig. 2).

Histological analyses showed the presence of newly formed tissue in nearly all defects, consisting of different structural components (Fig. 2). The newly formed tissue mainly consisted of homogenous distributed cells filling up the defect (regenerative tissue, RT). This regenerative tissue was covered by a multi/monolayer of elongated cells (ML) arranged parallel to the surface with a smooth appearance that was attached to the surface of the native cartilage tissue. In addition, thin and fibrous ECM structures were present, being part of the regenerative tissue, integrating with the top part of the native cartilage ECM. Cells of the regenerated tissue attached to the cartilage defect borders and cells were present within edges and fissures of the cartilage showing a high level of integration of regenerative tissue with native cartilage tissue (Fig. 2).

In the newly formed tissue, collagen type I (COL I) protein could not be detected by IHC analyses in most of the co-cultures, neither in the implanted spheroids nor in the regenerative tissue. Only in two samples, some COL I protein expression was observed in the implanted spheroids but only in the spheroid rims or in small areas within the spheroid (Additional file 1: Figure S1). Protein expression of collagen type II (COL II), the predominant collagen present in cartilage, was neither detected in the formed repair tissue nor in the implanted spheroids. However, in 12 of the 14 donor samples, the newly formed ECM fibers present at the defect borders showed COL II protein expression (Additional file 1: Figure S1).

**Fig. 3:**
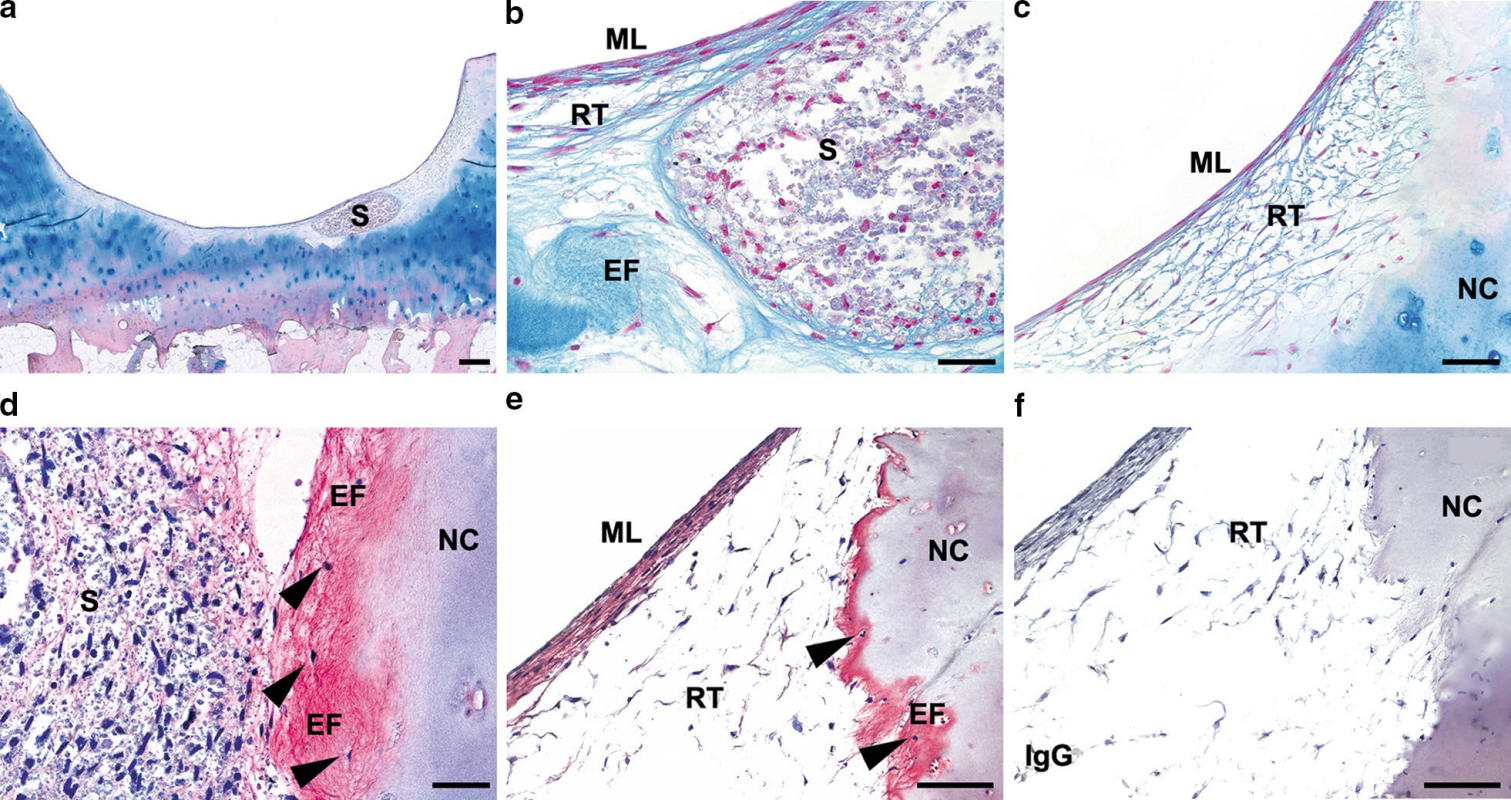
Proteoglycan protein expression in co-cultures.* Alcian blue* staining of a representative donor sample is shown (donor #15) (**a**–**c**). **a** An overview of a co-culture including one implanted spheroid (S) and (**b**) higher magnification of the implanted spheroid, surrounded by GAG positive ECM fibers (EF) (c) GAG containing regenerative tissue (RT) and multilayer (ML) at the surface.* Alcian blue* staining stains GAGs *blue*, and nuclear fast *red* stained the *nuclei red*. (**d**–**f**) Immunohistochemical detection of aggrecan protein expression shown as red staining of representative donor samples. ACAN protein expression in donor #8 (**c**) showing a low expression in implanted spheroids (S) and a high expression in ECM fibers (EF) connected to the native cartilage (NC). **d** ACAN protein expression in the ML of the regenerative tissue in donor #8, as well as in the ECM fibers. Single cells (*arrowheads*) were often detected within the ECM fibers. **e** IgG isotype control. Sections were counterstained with haematoxylin, staining the* nuclei blue*. *Scale bar* of **a**: 500 µm; **b–f**, 100 µm; *EF* ECM fibers, *ML* multilayer, *NC* native cartilage, *RT* regenerative tissue, *S* spheroid, *SB* subchondral bone

In addition to collagens, GAGs are important components of the cartilage ECM. GAGs can be considered as metabolites that are indicative for the potency of chondrocytes and can be visualized by Alcian blue staining of tissue sections. The implanted spheroids were positive for GAGs 12 weeks after co-cultivation with the condyle chips (Fig. 3a–c). In the present repair tissue, the top multi/monolayer of elongated cells was in general more intensively stained as the regenerative tissue below (Fig. 3b, c). Moreover, the previously described thin ECM fibers connected to the native cartilage tissue showed the presence of GAGs (EF in Fig. 3b), showing a stronger staining intensity as the regenerative tissue. GAGs are closely connected to the main proteoglycan in articular cartilage, ACAN. In the co-cultures, the implanted spheroids in almost all cartilage defects showed presence of ACAN protein after 12 weeks of co-cultivation, however, the area and location of ACAN positive regions varied between the donor samples. In the co-cultures, ACAN protein was detected in all of the different components of the regenerated tissue (Fig. 3d, e). Nearly all monolayer/multilayer of the co-cultures as well as the regenerative fibrous tissue were positive for ACAN (Fig. 3d, e), except for the co-cultures of donor #5 and #7. Moreover, ACAN protein was also present in the newly formed ECM fibers connected to the native cartilage tissue of all co-cultures, except of condyle chips implanted with spheroids of donor #5. Within the newly formed ECM fibers, the presence of single cells was observed (arrowheads in Fig. 3d, e). ACAN positive areas varied within the newly formed tissue, showing larger ACAN positive regions in top layers and in close proximity to implanted spheroids. Whereas co-cultures of 7 donor samples (donor #8, #9, #10, #11, #12, #16 and #17) showed high expression levels of ACAN, co-cultures of 3 donor samples (#5, #7 and #13) showed only few positive areas of ACAN protein in the regenerative tissue.

**Fig. 4:**
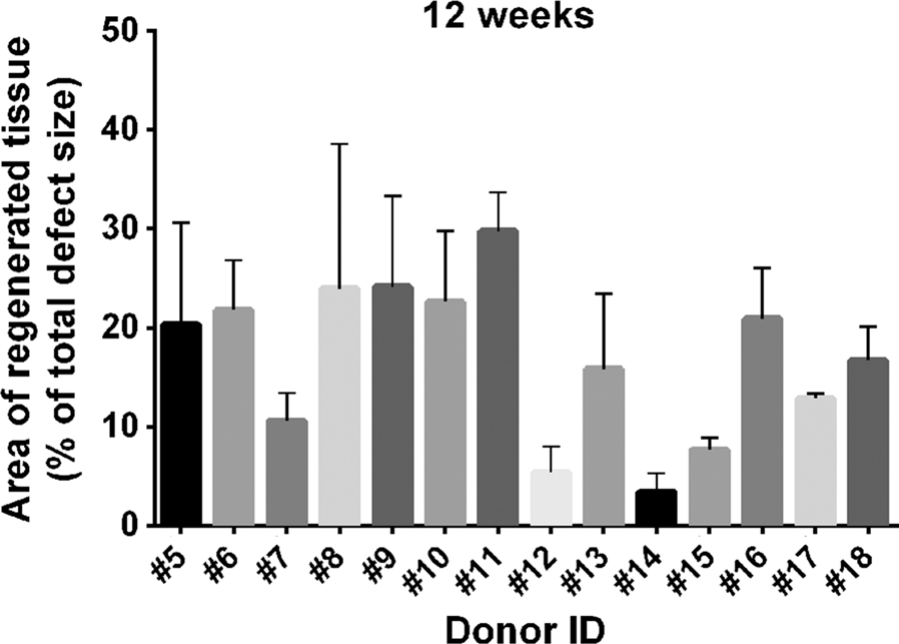
Histomorphometric analysis to determine the amount of repair tissue. Shown are the areas of newly formed tissue as percentage of the total defect area, excluding the area occupied by present spheroids as determined by histomorphometric measurements, after 12 weeks of co-cultivation. Indicated are the donor ID and the means and standard deviations of area measurements

The level of regeneration after 12 weeks of spheroid co-cultivation with condyle chips were assessed histomorphometrically, excluding the area occupied by the implanted spheroids (Fig. 4). Whereas, co-cultures of 7 donors (donor #5, #6, #8, #9, #10, #11 and #16) contained the highest amount of newly formed regenerative tissue filling-up around 20–30% of the defect area, co-cultures of 4 samples (donor #7, #13, #17 and #18) showed lower amounts of regenerated tissue of 10–16% of the total defect area (Fig. 4). Only 2 donors (donor #12 and #15) showed very little regenerative tissue filling-up 5–7% of the defect area, whereas regeneration of the cartilage defect by spheroids from donor #14 did not exceed the regeneration level found in the negative controls (Additional file 2: Figure S2 b). The used negative controls were condyle chips without any implanted spheroid, which also showed low levels of newly formed tissue within the defect. Detached parts of mono- and multilayer cells but no clear regenerative tissue and integration in the cartilage defect were observed (Additional file 2: Figure S2 a).

### Spheroid composition before implantation into human subchondral cartilage defects

**Fig. 5:**
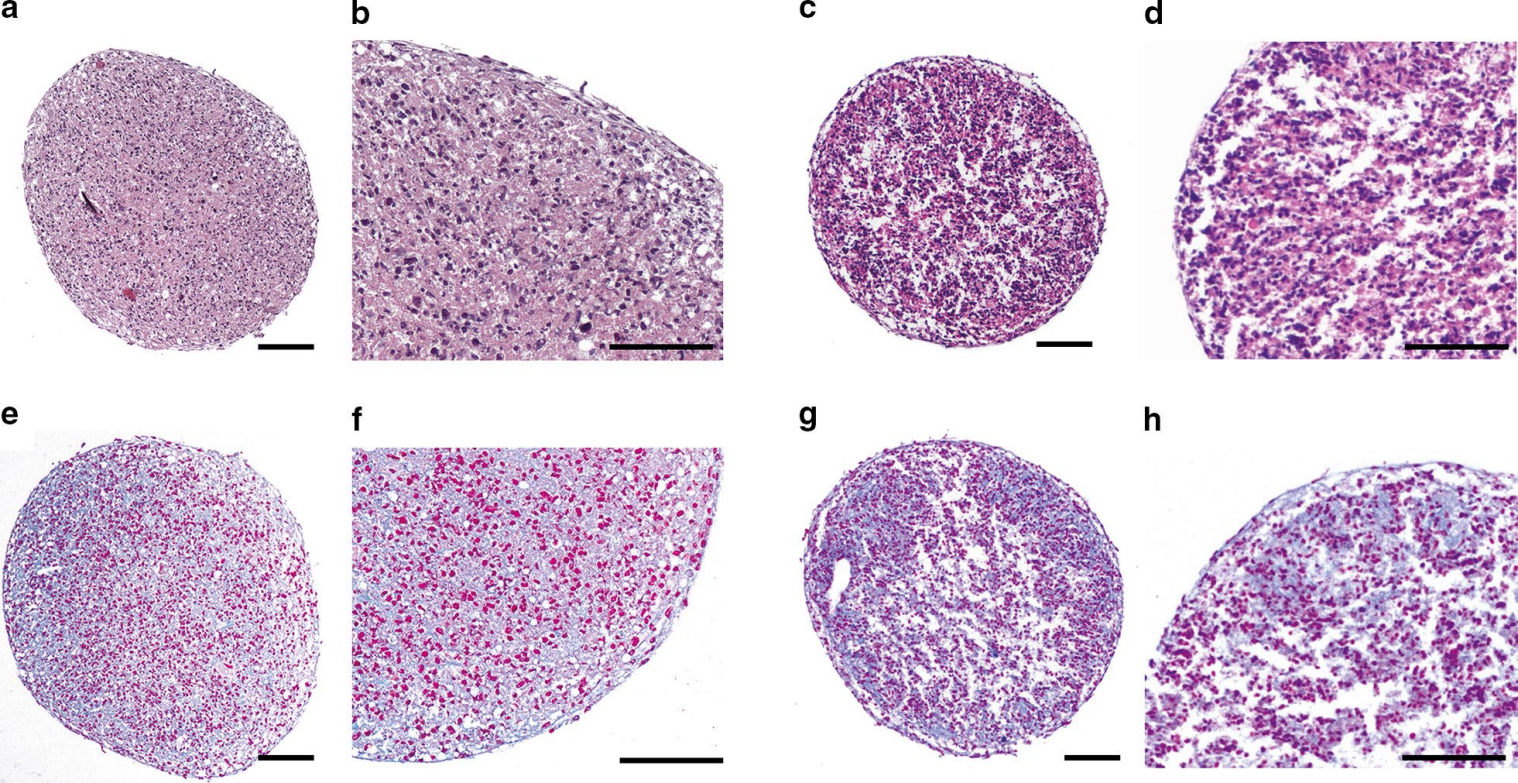
Histological appearance of 2 week old spheroids. Shown are two representative examples of HE (**a**–**d**) and *Alcian blue* (**e**–**h**) stained cross sections of spheroids after 14 ± 1 day of culture. HE staining of donor #9 (**a**, **b**) and #10 (**c**, **d**), with magnifications of **a** and **c** being shown in **b** and **d** respectively. *Alcian blue* staining of donor #9 (**e**, **f**) and #10 (**g**, **h**), with magnifications of **e** and **g** being shown in **f** and **h** respectively.* Alcian blue* stained the GAGs present in the ECM *blue*, whereas *nuclear fast red* stained the nuclei of the cells *red*. The samples were stained in different staining batches, causing a difference in observed staining intensity. *Scale bar* 100 µm

Spheroids derived from the same donor (same batch) as implanted in the condyle chips were also characterized before implantation to allow for a direct comparison of spheroid characteristics before implantation and their potency to regenerate tissue after implantation into the human subchondral cartilage defects. Spheroids derived from all donor samples showed a similar spheroid diameter at the time of implantation of 766.61 ± 167.33 µm as well as a similar morphology. The rims of the spheroids were composed of elongated cells whereas the cells in the center were round in shape as shown by HE staining for donor #9 and #10 (Fig. 5a–d). Alcian blue staining showed the presence of hyaline-specific glycosaminoglycans (GAG) within spheroids, which indicated chondrogenic activity of the cells (shown for donor #9 and #10 in Fig. 5e–h).

**Fig. 6:**
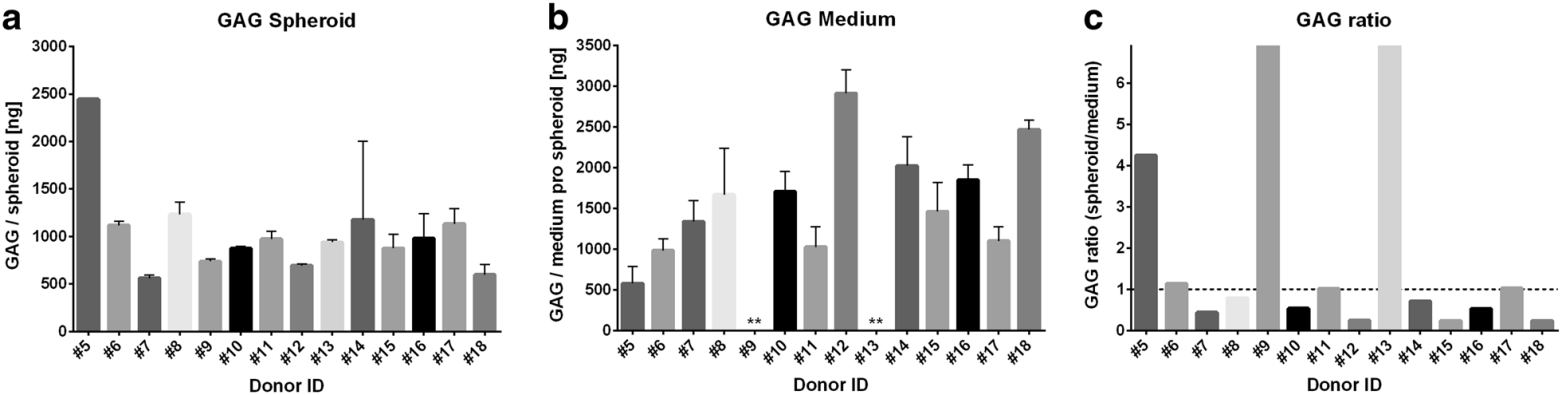
GAG produced per spheroid of individual donors. Produced sulfated GAGs per spheroid were quantitatively measured using the DMMB assay. **a** Amount of GAGs bound within the spheroids, as determined for three individual spheroids per donor. Only for donor #5, only one spheroid was analysed. **b** GAGs released into the medium per spheroid, as determined for the same three individual spheroids as the bound GAGs were determined for. **Donors #9 and #13 revealed negative values, indicating that GAGs were not detectable in medium. GAG was measured in spheroids of 14 ± 1 days, except for donor #14 and #18 where spheroids were cultivated for 24 days. Samples were measured in triplicates. Indicated are mean ± SD of single spheroids. **c** Calculated ratios of GAG bound within spheroids to GAG released into the medium per individual spheroid (Ratio GAG_bound_/GAG_released_). Dotted line indicates a ratio of 1.0, indicating an equal amount of bound and released GAG per spheroid. Ratios >1.0 indicates that the GAG content bound in the individual spheroids is larger than in media and ratios <1.0 indicates that the GAG content bound in spheroids is less than in media. For samples #9 and #13, no GAGs were measured in medium, and therefore the ratio of GAGs spheroid/medium could not be calculated and was set as infinite

Quantification of GAGs either bound in spheroids or released into the cell culture medium revealed donor-dependent variation in GAG production in the spheroids. The amount of GAGs bound in the spheroids ranged from 562.2 ng/spheroid to 2445.9 ng/spheroid (Fig. 6a). The amount of GAGs released into the cell culture medium showed a similar range of 580.5 ng/spheroid to 2919.7 ng/spheroid (Fig. 6b). For two samples no GAGs were detectable in the cell culture medium. The ratio of bound/released GAG per spheroid showed large variations between the individual donors, ranging from 0.24 to 4.2 with a ratio of >1 indicating more GAGs bound into the spheroid than released into the medium. In total, 8 out of 14 donors revealed a ratio below 1, thus releasing more GAG in medium than being bound into the spheroids, whereas 4 out of 14 samples contained more GAG bound into spheroids than released into the medium (Fig. 6c). Two donors showed approximately a similar GAG content bound into spheroids and released into the medium.

### Expression of chondrogenic markers in spheroids before implantation

The differences in production of hyaline specific GAG suggested a difference in potential of the spheroids to express cartilage specific molecules. To analyse the potential differences in expression of other cartilage specific molecules, the gene expression level of the chondrogenic genes *S100B*, *CRTAC1* (cartilage acidic protein 1) and *ACAN* (aggrecan) was analysed in the spheroids. Due to limited amount of material only 12 of 14 spheroid donor samples could be analysed for gene expression (spheroids from donor #9 and #13 were not analysed).

**Fig. 7:**
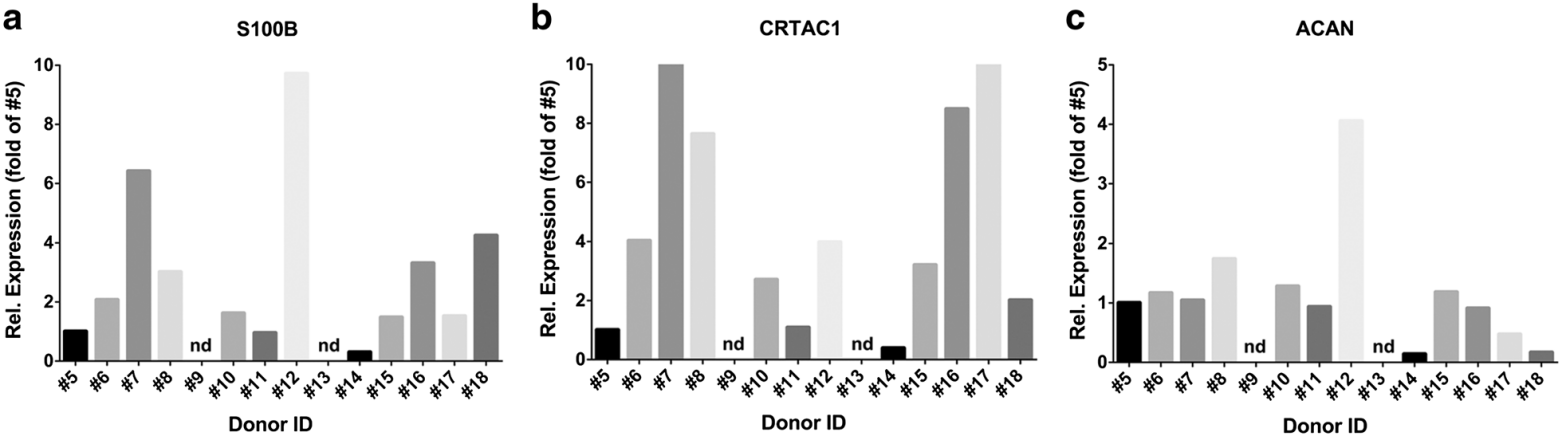
Gene expression analysis of chondrogenic markers. Relative gene expression of chondrogenic marker genes in the individual donor samples. **a** *S100B*, **b** *CRTAC1*, **c** *ACAN*. Sample #5 was set to 1 and the other samples were expressed relative to sample #5. Each sample was analysed in triplicates. Due to the low numbers of generated spheroids for donor #9 and #13, no spheroids were available (n.a.) for qPCR analysis of these samples. The calculation of gene expression levels was based on the ΔΔCt method. The gene expression levels were normalized to GAPDH gene expression levels

*S100B* is a transcriptional target of the chondrogenic specific *SOX* genes and expressed in early stages during chondrocyte differentiation [19]. The relative gene expression levels of *S100B* showed a large variability between the different donor samples, showing differences of up to 36-fold between the donor samples (Fig. 7a). Similar large differences in gene expression were observed for *CRTAC1*, which is a specific marker for cultured chondrocytes [20] and is also used as an indicator for redifferentiation of cells once they are transferred from a monolayer into a 3D cell culture system [21]. For *CRTAC1* differences up to 28-fold between the donor samples was shown (Fig. 7b). The main proteoglycan present in the articular cartilage ECM (extra-cellular matrix), *ACAN* [22], showed much less variation in gene expression levels between the donor samples as observed for *S100B* and *CRTAC1* gene expression. Only spheroids derived from one donor (donor #12) showed high *ACAN* gene expression levels, which were up to 2.5-fold higher compared to the other samples. In contrast, the spheroids of donor #14 and #18 showed very low *ACAN* expression levels compared to the other samples (Fig. 7c).

**Fig. 8:**
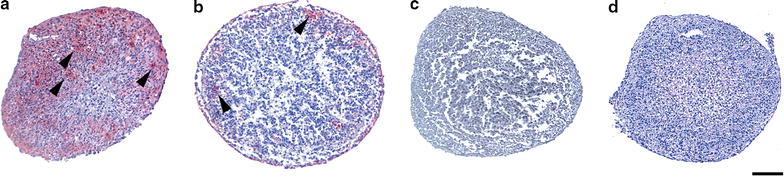
Aggrecan protein expression in 2 week old spheroids before implantation. Immunohistochemical detection of ACAN protein expression shown as a* red* staining (arrowheads) in representative spheroid samples before implantation. **a** High ACAN protein expression in spheroids of donor #8, **b** lower expression predominantly in the outer layers of spheroids of donor #10 and **c** absence of ACAN expression in spheroids of donor #14. **d** IgG isotype control. Sections were counterstained with haematoxylin, staining the *nuclei blue*. *Scale bar*: 100 µm

Although *ACAN* showed little variation in gene expression levels in the different donor samples, it is a predominant cartilage specific ECM protein and therefore its expression was also analysed at the protein level by immunohistochemical analyses. In contrast to the similar *ACAN* mRNA levels observed in the different donor samples, very different levels of ACAN protein expression within spheroids before implantation were observed (Fig. 8). Three donor samples showed high ACAN protein levels (largest areas of the spheroids showing presence of ACAN protein), with ACAN protein being present both in the center and outer layers of the spheroids (donor #8, #9 and #5) (Fig. 8a). Seven donor samples showed average ACAN protein levels, showing predominant expression in the outer layers of the spheroid (donor #11, #10, #6, #16, #17, #15 and #12) (Fig. 8b) and 3 donor samples showed a low level of ACAN protein expression, being present only in the outer layer of the spheroid (donor #13, #18 and #7). Interestingly, ACAN protein expression was absent in one donor, donor #14 (Fig. 8c), which showed no regeneration potential after 12 weeks of co-cultivation with a cartilage condyle chip (Additional file 2: Figure S2 b).

### Aggrecan protein expression in spheroids as potency marker for cartilage regeneration capacity

The aim of this potency study was to identify a specific characteristic of spheroids before implantation that would be a predictive marker for the regeneration potential of spheroids after implantation. The individual spheroid characteristics as described above were correlated to the level of newly formed repair tissue after 12 weeks of co-cultivation of spheroids into human cartilage defects (Table 1). To do so, the characteristics of the spheroids were scored and the results of all donor samples were used to form a ranking from high to low levels of the specific parameter. The same was done for the level of tissue regeneration after implantation. The two spheroid characteristics that showed a potential positive correlation with regeneration in the co-culture system were the production of GAGs, specifically the GAG ratio bound/released, and ACAN protein expression in spheroids before implantation (Table 1). The gene expression of *ACAN*, *CRTAC1* and *S100B*, despite large gene expression differences for *CRTAC1* and *S100B* in spheroids before implantation, as well as the general spheroid characteristics did not show a correlation to the tissue regeneration capacity of the spheroids after implantation using this correlation analyses.

**Table 1:** Ranking of donors according to regeneration capacity compared to the ranking of aggrecan protein expression and GAG ratio in co-cultures cultivated for 12 weeks

Donor ID	Area of regenerated tissue ± SD (%)	Area of regenerated tissue (ranking)	ACAN protein by IHC (ranking)	GAG_bound/released_ (ranking)
#11	29.76 ± 3.92	14	7	8
#8	23.96 ± 14.63	11	14	5
#9	24.16 ± 9.15	11	12	14
#10	22.60 ± 7.17	11	7	3
#6	21.78 ± 5.05	10	10	9
#16	20.91 ± 5.13	10	10	3
#5	20.32 ± 10.30	9	14	12
#13^a^	15.82 ± 7.63	7	5	14
#18^a,^ ^b^	16.73 ± 3.39	7	5	1
#17	12.83 ± 0.53	5	7	8
#7	10.59 ± 2.80	4	5	3
#15^a^	7.68 ± 1.17	3	7	1
#12	5.37 ± 2.61	2	10	1
#14^a,^ ^b^	3.41 ± 1.91	1	1	5

Shown is the comparison between the ranks of regeneration of each donor sample (ID) and the ranks of ACAN protein expression and GAG ratio in spheroids before implantation. The samples are ranked according to their level of tissue regeneration as percentage of defect filling. Assignment of ranks of ACAN protein expression as analysed by IHC analysis was based on presence of ACAN positive areas within spheroids where largest positive areas were assigned to the highest rank (14) and absence of ACAN protein was assigned to lowest rank (1). The results of the GAG ratio and their assigned rank are also indicated. Ratios >1.0 indicated that the GAG content bound within spheroids is larger than in media and ratios <1.0 indicates that the GAG content bound within spheroids is less than in media. High GAG ratios were assigned to high GAG ranks (rank: 10–14), low GAG ratios were assigned to low ranks (rank 1–4)

^a^Samples derived from the manufacturing department

^b^The duration of cultivation of spheroids until characterisation was 14 ± 1 days, except for donor #14 and #18, which were derived from the manufacturing department and obtained after culturing for 24 days

Spheroids with high ratios of GAG bound within spheroids to GAG released into cell culture medium displayed in general a good regeneration capacity, with more GAGs being bound within the spheroid than released into the medium (Table 1). Of the seven samples that showed a high level of tissue regeneration (donor #5, #6, #8, #9, #10, #11 and #16), 4 samples showed a high GAG ratio (donor #5, #6, #9 and #11). The remaining samples showed either an average GAG ratio (donor #8) or a low GAG ratio (donor #10 and #16). In contrast, of the five samples showing a low level of regeneration (donor, #7, #12, #14, #15 and #17), 4 samples showed a low GAG ratio (donor #7, #12 #14 and #15) whereas only donor #17 showed a high GAG ratio. This suggested a trend towards a positive correlation of tissue regeneration and GAG production by spheroids, as the majority of the samples that showed a high GAG_bound/released_ ratio showed a good tissue regeneration. In contrast, the majority of the samples showing a low GAG_bound/released_ ratio showed a poor tissue regeneration.

Spheroids showing a high ACAN protein expression (as scored by large positively stained areas), showed a better regeneration capacity compared to the spheroid samples showing low expression levels of ACAN protein (only few positively stained areas) before implantation (Table 1). Of the seven donor samples that showed the highest level of tissue regeneration (donor #5, #6, #8, #9, #10, #11, #16), five samples showed also high ACAN protein expression (donor #5, #6, #8, #9 and #16) whereas two samples (donor #10 and #11) showed average ACAN protein levels before implantation. Of the five donor samples with a low tissue regeneration level (donor, #7, #12, #14, #15 and #17), two samples showed low ACAN protein levels (donor #7 and #14), two samples average levels (donor #15 and #17), whereas one sample showed high levels (donor #12). Most importantly, in the donor sample #14 that showed the lowest tissue regeneration and did not exceed the level of regeneration of the negative control (3.41% of regeneration), ACAN protein was completely absent (Table 1).

To confirm the observed potential correlations between spheroid characteristics before implantation and regenerative capacity after implantation, statistical analysis using the Spearman’s correlation test for nonparametric variables was performed. Although a potential positive correlation of the production of GAGs in spheroids, specifically the GAG ratio bound/released, and tissue regeneration was observed using the ranking system (Table 1), after calculating the Spearman’s correlation coefficient between these two variables, no significant correlation was found between GAG ratio and tissue regeneration levels (r = 0.4087; p < 0.075) but only a trend (Table 2). For the gene expression of the chondrogenic markers *S100B*, *CRTAC1* and *ACAN*, no correlation was found with tissue regenerative capacity as analysed using Spearman’s correlation coefficient (Table 2).

**Table 2:** Correlation between spheroid characteristics and tissue regeneration capacity

Spheroid characteristics	Correlation with level of tissue regeneration and statistical significance
ACAN protein	r = 0.55; p < 0.025
ACAN mRNA	r = 0.53; p < 0.05^a^
GAG spheroids	ns
GAG medium	ns
GAG ratio	r = 0.41; p = 0.073
S100BmRNA	ns
CRTAC1 mRNA	ns

Spearman’s correlation coefficient r was calculated between spheroid characteristics analysed before implantation and the capacity of tissue regeneration after 12 weeks of co-cultivation in an ex vivo cartilage repair model

*ns* not significant

^a^ACAN mRNA levels do correlate with tissue regeneration when one statistical outlier (#12) with extremely high ACAN mRNA levels is taken out of the analysis

**Fig. 9:**
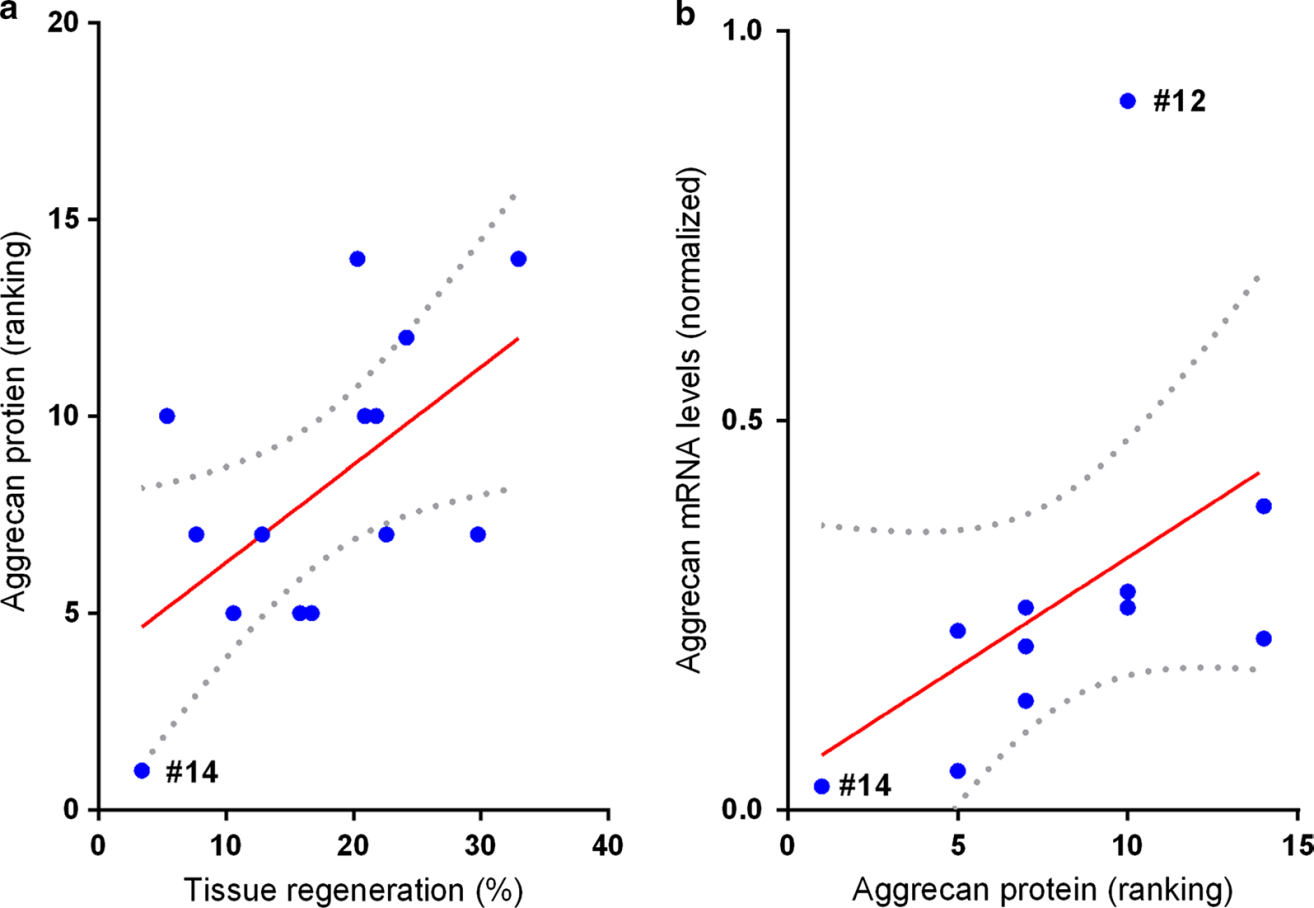
Linear regression between aggrecan expression in spheroids and tissue regeneration capacity. Statistical dependency between chondrocyte spheroid characteristics and tissue regeneration capacity was tested by calculating the Spearman’s correlation coefficient (see Table 2). **a** Linear regression of tissue regeneration and ACAN protein expression in spheroids before implantation. Goodness-of-fit R_2_ = 0.347 (p < 0.03). **b** Linear regression of tissue regeneration and *ACAN* mRNA levels. Since spheroids derived from donor ID12 display extremely high mRNA levels it was defined as a statistical outlier in this analysis. For the rest of the patients, tissue regeneration is significantly correlated with tissue regeneration

As was already indicated by the correlation analyses using the ranking system (Table 1), statistical analysis using the Spearman’s correlation test between tissue regeneration and ACAN protein expression in spheroids before implantation indeed revealed a moderate correlation (r = 0.55; p < 0.025) (Table 2). Linear regression analysis (Fig. 9), clearly distinguished donor sample #14 that showed the lowest tissue regeneration that did not exceed the level of regeneration observed in the negative control, from the rest of the samples. No significant correlation was found between tissue regeneration and *ACAN* mRNA levels as described above, which was due to a statistical outlier that showed low tissue regeneration but very high *ACAN* mRNA levels (donor #12). Of note, when taking out #12, a positive correlation was found between tissue regeneration and *ACAN* mRNA levels using Spearman’s correlation test (r = 0.53; p < 0.05). Moreover, a high correlation (r = 0.68; p < 0.009) was found between *ACAN* mRNA levels and ACAN protein expression. Again, donor #12 is considered an outlier because of its high ACAN protein and mRNA levels compared to the rest of the spheroids, which is clearly visible in the linear regression analysis as shown in Fig. 9.

## Discussion

Cell therapeutics to treat cartilage defects include autologous chondrocyte implantation products. However, little is known on the correlation of cartilage cell transplant properties before implantation and their potency to regenerate cartilage tissue after implantation. In this study, an ex vivo human cartilage repair model was developed, consisting of human condyle chips in which a standardized subchondral cartilage defect was manually set, being representative of cartilage defects as treated in the clinic. This model was used to test the potency of a cartilage cell transplant. To do so, cartilage cell transplants (spheroids) were implanted into these defects in a clinical relevant dosage and the defect filling and tissue regeneration process was followed ex vivo for 12 weeks. Most importantly, before implantation, characteristics of spheroids from the same batch as used for the implantation were determined with respect to general spheroid characteristics, gene expression of the chondrogenic marker genes *CRTAC1*, *ACAN* and *S100B* as well as the production of extra-cellular matrix components (GAGs, glycosaminoglycans) and correlated with the potency of this batch to fill up the defect. The aim of this potency study was to select a specific characteristic of spheroids that would be a predictive marker for the regeneration potential of spheroids after implantation.

Similar ex vivo cartilage repair models to study the mode-of-action of cell transplants have been performed previously. In an osteochondral in vitro bovine model using cartilage derived from calves, the tissue regeneration capacity of bovine chondrocytes was analysed [16]. In this model, the best tissue regeneration was observed in the osteochondral defects, compared to the chondral and subchondral defects and hyaline-like tissue was formed, as shown by Safranin-O staining. The same model was used to analyse the repair capacity of human bone-marrow derived stem cells (hBMSC) in an explant model, which also formed hyaline-like repair tissue [16]. The amount of regenerated tissue in this explant model were very similar to the observed degree of regeneration in the ex vivo human condyle chip model presented in this study. Moreover, nearly similar results were shown in an in vivo cartilage regeneration model, where treatment of osteochondral defects with a hydrogel in rabbits resulted in the formation of less than 25% fibrocartilage-like repair tissue after 24 weeks [23]. This confirms the suitability of the ex vivo human cartilage repair model presented in this study to assess cartilage repair capacity by cartilage cell transplants. However, most importantly, this is the first time this was shown in a human model system. Although this is a suitable model to evaluate potency of cartilage cell transplants, the use of these type of cartilage chips with implanted spheroids and subsequent implantation in an explant mouse model showed an enhanced integration and differentiation of the repair tissue [7] compared to the ex vivo model in this study. However, both studies showed the capacity of the spheroids to form a hyaline-like repair tissue in cartilage defects.

The analyses of the co-cultures after 12 weeks of co-cultivation confirmed that spheroids derived from different donor samples displayed different biological activities, observed as a different degree of tissue regeneration by the spheroids in the artificial cartilage defect. Donor samples differed in age and gender but there were no obvious indications that this affected the potency of spheroids and confirmed observations in clinical practice where no significant differences in regeneration of condyle defects in male and female donors was observed [24]. The newly formed tissue mainly consisted of homogenous distributed cells filling up the defect, which was covered by a multi/monolayer of elongated cells arranged parallel to the surface with a smooth appearance attached to the surface of the native cartilage tissue. In addition, thin and fibrous ECM structures were present, being part of the regenerative tissue, integrating with the top part of the native cartilage ECM. Cells of the regenerated tissue attached to cartilage defect borders and cells were present within edges and fissures of the cartilage showing a high level of integration of regenerative tissue with native cartilage tissue. Hyaline-specific proteins were present in the newly formed tissue, to a different degree in the different tissue components of the regenerated tissue as well as to a different degree in the different donors. The presence of the proteoglycan ACAN and GAGs confirmed the hyaline-like character of the newly formed tissue, which is most likely not in a fully mature state yet as suggested by the absence of COL II. Moreover, in some samples, COL II protein was present in the newly formed ECM fibers. In contrast, COL I protein was not detected in the newly formed tissue, suggesting that the newly formed tissue did not contain dedifferentiated chondrocytes.

One of the structures being formed and part of the regenerated tissue were thin ECM fibers connected to native cartilage tissue, which differed in density, length and location between the different donors. It has been suggested that single cells that synthesize ECM fibers by extending native ECM with new matrix substances such as ACAN, require cell–matrix interactions [25]. Indeed, within these ECM fibers, single cells were located in close proximity and aligning the native cartilage tissue, suggesting that these cells synthesized these ECM fibers. In contrast to the neighboring native cartilage tissue, these newly formed ECM fibers contained large amounts of GAGs and ACAN protein, indicating new synthesis of these structures during the co-cultivation. This suggested that the cells that produce these ECM fibers are derived from the implanted spheroids. No ECM fibers were present in locations where no cells were attached to the native cartilage tissue confirming the requirement of implanted cells to synthesize these ECM fibers. These types of structures have not been reported previously.

The aim of this study was to select specific characteristics of spheroids before implantation that would be a predictive marker for the regeneration potential of spheroids after implantation. Neither general spheroid characteristics as spheroid size or cellularity, nor the gene expression of the chondrogenic markers *ACAN*, *CRTAC1* or *S100B* were a predictive marker for the potency of the cartilage cell transplant. These spheroid characteristics did not correlate to the level of tissue regeneration in the condyle chip model after co-cultivation for 12 weeks, as shown using the Spearman’s correlation test. Although it was shown that spheroids of different donor samples produce different amounts of GAGs and the ratio of bound to released GAG (per spheroid) showed a trend of a positive correlation with tissue regeneration capacity of spheroids, this could not be confirmed by Spearman’s correlation test that only showed a trend and not a statistical significant correlation.

In contrast, ACAN protein levels in spheroids before implantation showed clear differences between donor samples and showed a positive correlation with the level of formed repair tissue as shown by Spearman’s correlation test. Spheroids showing a high ACAN protein expression before implantation showed a better regeneration capacity compared to spheroid samples showing low expression levels of ACAN protein. Most importantly, in the sample that showed the lowest tissue regeneration level no ACAN protein was present before implantation. Although no significant correlation was found between tissue regeneration and ACAN mRNA levels, this was most likely due to a statistical outlier that showed low tissue regeneration but very high ACAN mRNA levels (donor #12). Removing sample #12, also a positive correlation was found between tissue regeneration and ACAN mRNA levels using Spearman’s correlation test. Moreover, a high correlation between ACAN mRNA levels and ACAN protein expression was observed. Altogether, this suggested that ACAN protein synthesis is directly involved in regenerative potency of spheroids. This is supported by the observed positive correlation of ACAN expression and potency in other cartilage cell transplant [8, 26].

The positive correlation of high ACAN protein expression and potency of spheroids suggests that ACAN protein levels determine the potency of spheroids to regenerate tissue within cartilage defects after implantation. The level of ACAN protein expression is indicative for the differentiation state of chondrocytes [27, 28]. An increased differentiation state of chondrocytes results in a higher production of ACAN protein. This suggested that chondrocytes that are in a more differentiated state have a higher regenerative capacity as less differentiated chondrocytes. Limitation of the study however is the lack of a direct correlation of the ex vivo potency assay to clinical performance and tissue regeneration in patients. This was due to the fact that the clinical studies and the condyle chip assay were performed separately. It would be important to confirm the correlation of ACAN expression and potency of spheroids in a clinical setting to confirm the proof-of-concept and predictive ability for potency of this ex vivo model system.

## Conclusions

The spheroid characteristic ACAN protein expression is a promising parameter to predict the regenerative capacity of spheroids and can potentially be used as a surrogate potency assay. In summary, the observed correlations of the spheroid characteristics with tissue regeneration capacity would fulfill the general requirements of a potency test. It would allow for the quantitative measurement of a biological activity associated with indication-specific functions of the product [10]. Analysis of ACAN protein expression can be performed quantitatively and moreover is associated with articular hyaline cartilage regeneration.

## Additional files

**Additional file 1: Figure S1.** Collagen type I and II protein expression in co-cultures. (**a–d**) Immunohistochemical detection of collagen type I protein expression shown as red staining in representative donor samples (**a**) slightly positive collagen type I staining (arrowhead) within implanted spheroid of donor #5 and (**b**) collagen type I protein expression in the superficial layer (arrow) of native cartilage of the co-culture of donor #15 (**c**) collagen type I expression in the subchondral bone (SB). (**d**) IgG isotype control (negative control). (**e–g**) Immunohistochemical detection of collagen type II protein expression shown as red staining in representative donor samples (**e**) co-culture of donor #5, revealing collagen type II expression in the ECM fibers connected to the native cartilage tissue (arrowhead). (**f**) Collagen type II protein expression in native cartilage (NC) of donor #13. (**g**) IgG isotype control (negative control). Sections were counterstained with haematoxylin, staining the nuclei blue. Scale bar of A, B: 50 µm; C, D: 250 µm; E: 100 µm; F, G: 500 µm. ML, monolayer; NC, native cartilage; RT, regenerated tissue; S, spheroid; SB, subchondral bone.

**Additional file 2: Figure S2.** Lack of regeneration potential in spheroids derived from patient #14. Histological appearance of repair tissue formed after 12 weeks as shown by HE staining of cross sections of a condyle chip. (**a**) Without implanted spheroids (negative control), where only a multilayer (ML) of cells is present. (**b**) Of a condyle chip with implanted spheroids of patient #14, where the spheroids are only covered by a newly formed multilayer of cells (ML) that does not exceed the regeneration level of the negative control in (**a**). Scale bar 500 µm. ML, multilayer; NC, native cartilage; S, spheroid; SB, subchondral bone.

## Abbreviations

ACI: autologous chondrocyte implantation
ECM: extra-cellular matrix
GAG: glycosaminoglycans
hBMSC: human bone marrow stem cells
OA: osteoarthritis
ACAN: aggrecan
CRTAC1: cartilage acidic protein 1
COL: collagen
