# Supplementary figures and images for: An ex vivo human cartilage repair model to evaluate the potency of a cartilage cell transplant
Source: J Transl Med. 2016 Nov 15;14:317. doi: 10.1186/s12967-016-1065-8 (PMC5111252; doi:10.1186/s12967-016-1065-8)

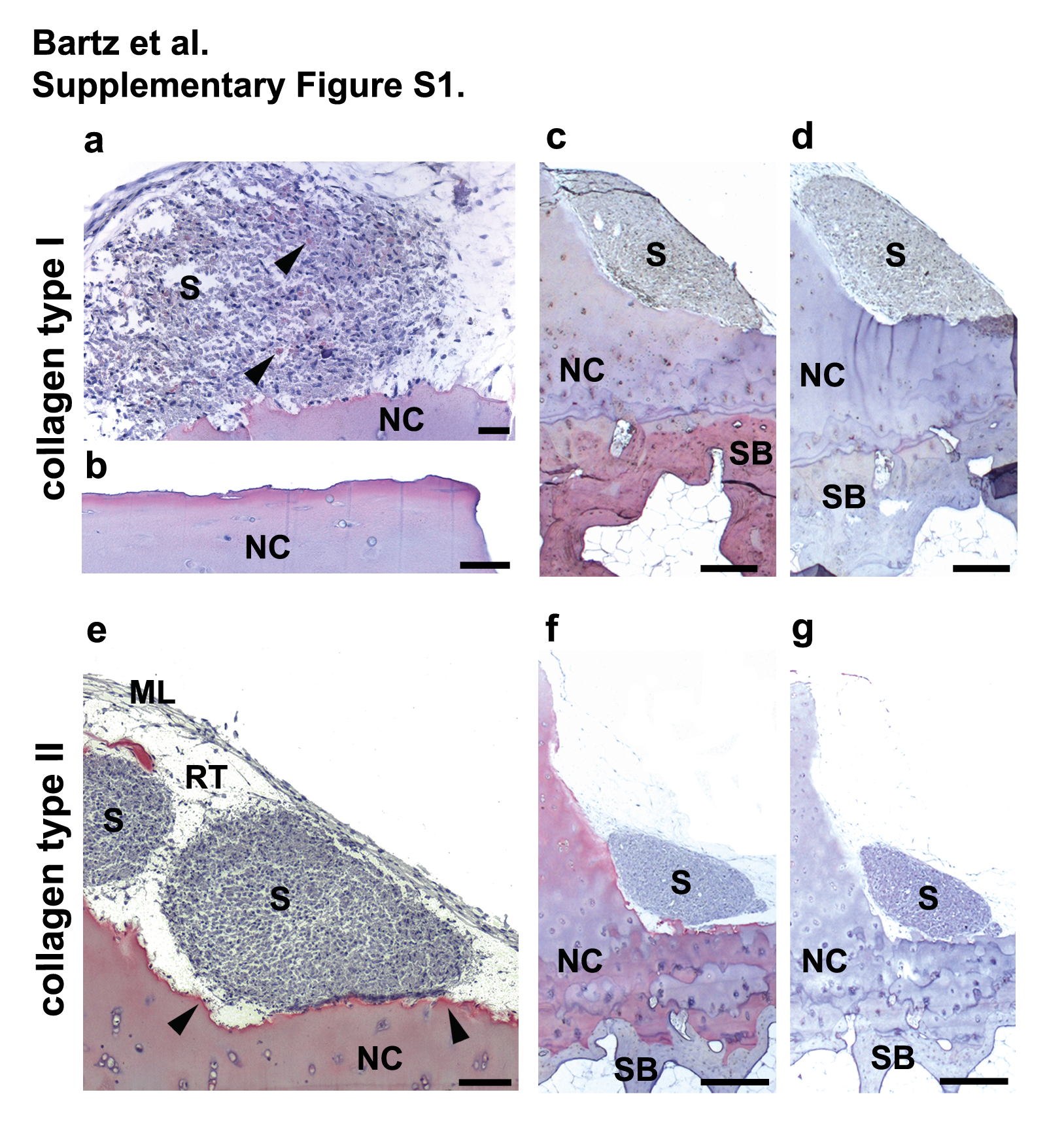

Supplement: Supplementary file 1 — Additional file 1: Figure S1. Collagen type I and II protein expression in co-cultures. (a–d) Immunohistochemical detection of collagen type I protein expression shown as red staining in representative donor samples (a) slightly positive collagen type I staining (arrowhead) within implanted spheroid of donor #5 and (b) collagen type I protein expression in the superficial layer (arrow) of native cartilage of the co-culture of donor #15 (c) collagen type I expression in the subchondral bone (SB). (d) IgG isotype control (negative control). (e–g) Immunohistochemical detection of collagen type II protein expression shown as red staining in representative donor samples (e) co-culture of donor #5, revealing collagen type II expression in the ECM fibers connected to the native cartilage tissue (arrowhead). (f) Collagen type II protein expression in native cartilage (NC) of donor #13. (g) IgG isotype control (negative control). Sections were counterstained with haematoxylin, staining the nuclei blue. Scale bar of A, B: 50 µm; C, D: 250 µm; E: 100 µm; F, G: 500 µm. ML, monolayer; NC, native cartilage; RT, regenerated tissue; S, spheroid; SB, subchondral bone. [file 12967_2016_1065_MOESM1_ESM.tif]

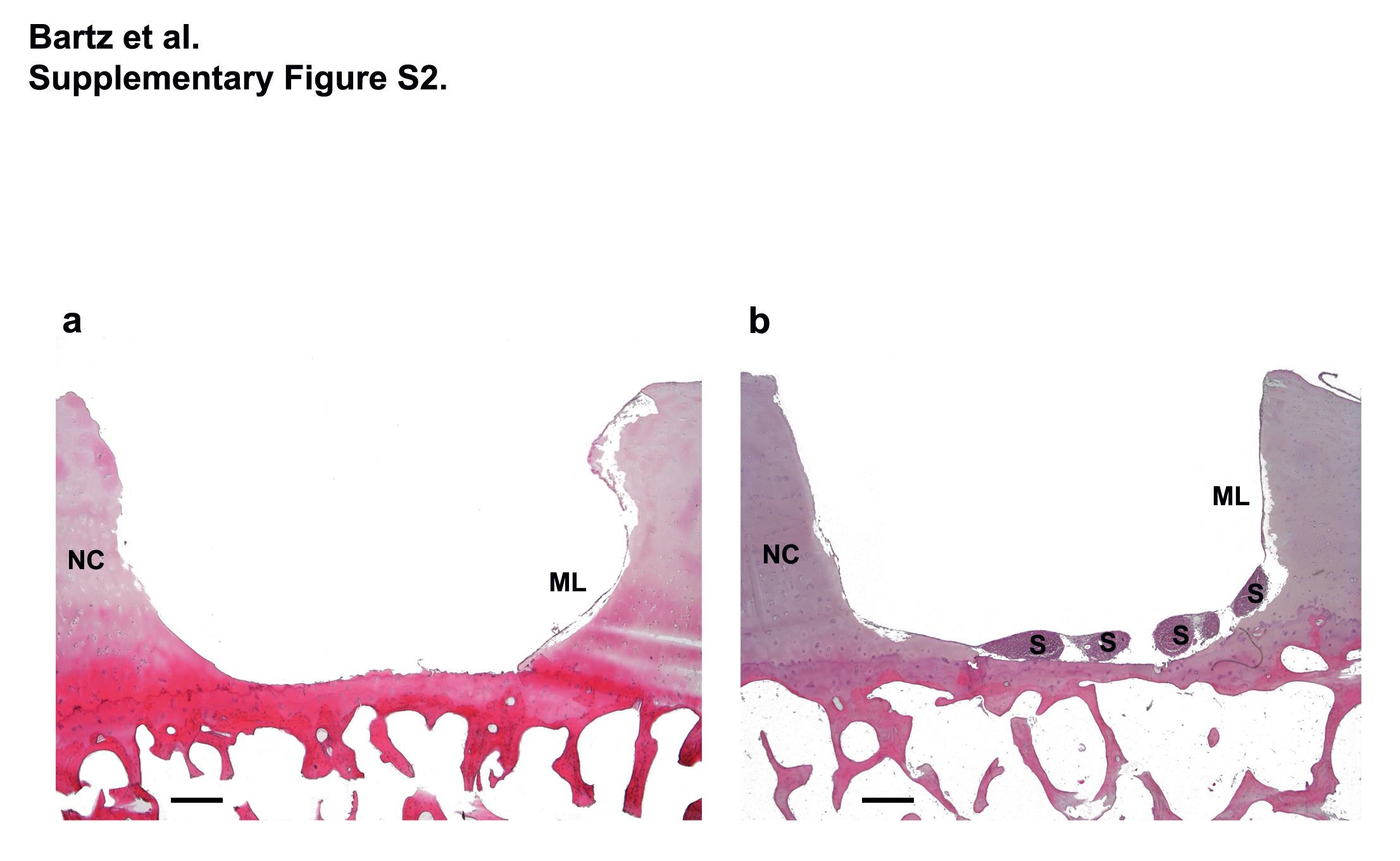

Supplement: Supplementary file 2 — Additional file 2: Figure S2. Lack of regeneration potential in spheroids derived from patient #14. Histological appearance of repair tissue formed after 12 weeks as shown by HE staining of cross sections of a condyle chip. (a) Without implanted spheroids (negative control), where only a multilayer (ML) of cells is present. (b) Of a condyle chip with implanted spheroids of patient #14, where the spheroids are only covered by a newly formed multilayer of cells (ML) that does not exceed the regeneration level of the negative control in (a). Scale bar 500 µm. ML, multilayer; NC, native cartilage; S, spheroid; SB, subchondral bone. [file 12967_2016_1065_MOESM2_ESM.tif]
